# Modelling Gaucher disease progression: long-term enzyme replacement therapy reduces the incidence of splenectomy and bone complications

**DOI:** 10.1186/s13023-014-0112-x

**Published:** 2014-07-24

**Authors:** Laura van Dussen, Marieke Biegstraaten, Marcel GW Dijkgraaf, Carla EM Hollak

**Affiliations:** 1 Department of Internal Medicine, Division of Endocrinology and Metabolism, Academic Medical Center, Amsterdam, 1100 DD, The Netherlands; 2 Clinical Research Unit, Academic Medical Center, Amsterdam, 1100 DD, The Netherlands

## Abstract

Long-term complications and associated conditions of type 1 Gaucher Disease (GD) can include splenectomy, bone complications, pulmonary hypertension, Parkinson disease and malignancies. Enzyme replacement therapy (ERT) reverses cytopenia and reduces organomegaly. To study the effects of ERT on long-term complications and associated conditions, the course of Gaucher disease was modelled.

The cohort consisted of all diagnosed GD patients in the Netherlands. Mutually exclusive disease states were defined as ‘asymptomatic’, ‘signs/symptoms’, ‘recovery’, ‘splenectomy’, ‘bone complication’, ‘multiple complications’ and ‘malignancy’. A natural history (NH) cohort was delineated based upon historical data on Dutch patients before ERT was available. Cumulative incidence curves were composed for progression from each disease state to the next. Two scenarios were applied for the ERT cohort: time to complications was calculated from A. start of ERT; B. entering the previous disease state.

Median time for the development of signs and/or symptoms was 30.1 years (N = 73). In the NH cohort (N = 42), 9% had developed a bone complication after 10 years in the signs/symptoms phase, while 21% had undergone a splenectomy. In the ERT cohort (N = 29 (A), N = 28 (B)), 12% (A) or 4% (B) had developed a bone complication after 10 years in this phase and no patient was splenectomized. No patients in the NH cohort recovered, compared to 50% in the ERT cohort after 3.6 years (N = 28 (A)) or 22.4 years (N = 27 (B)) of treatment. Median time from a first to a second complication was 11 years in the NH cohort (N = 31), whereas 16 respectively 14 percent had developed a second complication after 10 years in the ERT cohort (N = 17, scenario A/B). Fourteen percent (scenario A/B) developed an associated malignancy after 10 years in the phase ‘multiple complications’ (N = 23). Associated malignancies occurred almost exclusively in advanced disease stages, therefore it is suggested that ERT reduces their incidence

Long-term ERT for GD can reduce the incidence of splenectomy and bone complications. As ERT prevents progression to more advanced stages of GD it will most likely result in a reduction of associated malignancies.

## Background

Gaucher disease (GD; OMIM#230800) is an autosomal recessively inherited lysosomal storage disorder. GD results from a deficiency of the lysosomal enzyme glucocerebrosidase (or acid β-glucosidase, EC 3.2.1.45). The enzyme is encoded on chromosome 1 (1q21) and as a consequence of the deficiency storage of its substrate, glucocerebroside, occurs in macrophages. These lipid laden cells are called Gaucher cells and are primarily found in liver, spleen and bone marrow. GD type 1 is most common in the Ashkenazi Jewish population with an estimated birth prevalence of 1 per 855 [1]. The overall frequency of GD is much lower with an estimated prevalence in the population of 1 per 100.000. This can be an underestimation since several patients may remain undiagnosed. Three types of GD have been described. Type I GD (GD I), the most common phenotype, can be distinguished from the more severe types II and III GD based on the absence of the typical neurologic manifestations associated with the latter two forms. Clinical manifestations of GD I are highly variable, and the result of the accumulation of Gaucher cells in the liver, spleen and bone marrow compartment, leading to cytopenia, organomegaly and bone disease. Bone disease in turn, may present itself as atypical bone pain, severe bone crises or aseptic osteomyelitis, osteonecrosis, pathological fractures or vertebral collapse.

The course of GD including the age of onset of signs and/or symptoms may be influenced by precipitating factors i.e. Epstein-Barr virus infections or pregnancy [1],[2].

Long-term complications and associated conditions of GD I include liver complications with fibrosis or cirrhosis, an increased risk of associated malignancies including multiple myeloma and hepatocellular carcinoma [3],[4], Parkinson disease [5],[6] and pulmonary hypertension [7],[8]. Up until the early nineties, treatment of GD consisted purely of supportive measures such as blood transfusions in cytopenic patients and orthopaedic procedures in case of bone complications. Splenectomy was the only possible intervention in case of severe splenomegaly and cytopenia. However, it soon became apparent that splenectomy had a negative impact on bone involvement and accumulation of Gaucher cells in the liver [4],[9]-[12].

Since the early nineties therapy has become available in the form of purified enzyme, placental derived alglucerase later replaced by recombinant imiglucerase (Genzyme Corp, MA, USA) for the treatment of GD I and visceral manifestations of GD III. In 2002 substrate reduction therapy in the form of miglustat (Actelion Therapeutics) received marketing authorization. Miglustat is an inhibitor of glucosylceramide synthase and received authorization for the treatment of mild to moderate Gaucher disease. In 2010 velaglucerase alfa, another enzyme preparation (Shire Human Genetic Therapies, MA, USA) was approved for long-term treatment of GD I. New therapies are still emerging as taliglucerase alfa, yet another recombinant enzyme (Protalix, Biotherapeutics, Carmiel, Israel) has completed phase 3 clinical trials and has received authorization in the USA and Israel. An alternative, oral inhibitor of glucosylceramide synthase, eliglustat tartrate, is currently in the late stages of clinical development [13],[14].

Enzyme replacement therapy has been shown to be highly effective in reversing cytopenia and reducing organ volumes (see for example [15]-[21]). Controversy exists regarding its effect on bone disease, as this compartment seems slower to respond and skeletal disease may be partially unresponsive to therapy especially in a patient with a history of extensive bone manifestations before the initiation of treatment [22].

Besides these effects, beneficial effects on quality of life have been demonstrated [23]-[27]. Both substrate reduction therapy and enzyme replacement therapy are extremely costly with variations in costs depending on the agent and dose. Variations in dose exist between countries. Median dose in the Netherlands is 30 U/kg/month (range 15–120 U/kg/month), but doses up to 120 U/kg/m are prescribed internationally.

So far, cost-effectiveness analyses have not been performed. Connock addressed cost-effectiveness of several enzyme replacement therapies and concluded that the available data were insufficient to draw a conclusion [28]. The main limitations were the absence of natural history data and reliable information on quality of life. Real life experience with enzyme replacement therapy, mainly imiglucerase, suggests that when started early, i.e. before irreversible damage has occurred, ERT may prevent both the the need for splenectomy and the incidence of bone disease. Whether long-term complications and associated conditions of GD I can be prevented is debated [12],[29]. This is surprising, because ERT is already available in Western countries for 20 years. To address the issue of effects of ERT on long-term complications and associated conditions, we studied the progression of Gaucher disease, which can be used in a state transition model to assess the cost-effectiveness of Gaucher disease treatments. Such model is reported elsewhere in this journal [30].

## Patients and methods

This study was part of the TIPharma project T6-208: Sustainable Orphan Drug Development through Registries and Monitoring.

The Academic Medical Center is a national referral center for patients with Gaucher disease. This cohort consisted of all registered Gaucher disease patients in the Netherlands with a definite diagnosis of Gaucher disease based upon analysis of enzymatic activity and mutation analysis. Historical data were collected from all patients for whom a medical record before ERT was available (April 1991). For all patients who started ERT in the Netherlands after April 1991, prospective data were collected up to September 1, 2011.

### Patient cohorts

A natural history cohort and an enzyme replacement therapy (ERT) cohort were defined as follows:

#### NH cohort

Enzyme replacement therapy became available in the Netherlands in April 1991. Historical data on the progression of disease were retrieved from clinical records in all 90 patients. For the assessment of the time to onset of signs and/or symptoms the entire AMC cohort was analyzed, since only symptomatic patients are treated.

For the assessment of each consecutive disease stage only patients who had reached a particular disease stage before the era of ERT were included in the analysis. The reason for this is that inclusion of untreated patients after ERT became available would lead to an underestimation of disease progression as a result of bias by indication, as these patients exhibit milder disease manifestations. Thus, patients who had reached a particular disease stage prior to April 1991 were included in the analysis of the natural course of progression from that particular disease stage to the next, e.g. from signs and/or symptoms to bone complications. If a patient showed no progression before April 1991, then April 1991 was noted as the end of follow-up and the case was censored.

#### ERT cohort

Prospective data were collected from patients who were treated with ERT at the AMC (n = 64). In addition, the analysis included data from 2 patients who started treatment with ERT elsewhere. The data were retrieved from the start of ERT up to September 2011. Patients who switched from ERT to SRT (N = 2) data were included until the moment of switch. In patients who started treatment with substrate reduction therapy (N = 3) data were only included for the analysis of the natural course of disease (until the moment treatment with SRT was commenced). All patients have suffered from the worldwide imiglucerase shortage (June 2009). No distinction was made between patients who switched to treatment with either velaglucerase alfa or taliglucerase alfa as a result of the shortage. In addition, the consequences of dose reductions as a result of the shortage were not taken into account, as signs of deterioration were short-lived and did not affect the assignment of patients to categories of disease severity.

### Definition of disease states

To model the course of Gaucher disease, mutually exclusive disease states were defined as follows:

#### Signs/Symptoms

Most patients had developed signs and/or symptoms prior to the start of follow-up at the Academic Medical Center in 1991 or later. A record of signs/symptoms, cytopenia or organomegaly in the medical history was used as a criterion for the signs/symptoms stage. Patient files were scanned for any record of increased bleeding tendency, abnormalities in blood counts and/or organomegaly either upon physical examination or imaging. The date of onset of signs and/or symptoms was recorded as exactly as possible. If only the year in which signs and/or symptoms had occurred was recorded, the date was set for the first of January of that year. If only the age was mentioned, the date was set for the first of January of the year in which a patient reached that particular age.

#### Splenectomy

If a patient was splenectomized, the date at which a splenectomy had been performed was recorded. If only the month and year in which a patient was splenectomized were reported the date was set for the first of that specified month. If only the year in which a patient was splenecomized was recorded the date was set for the first of January of that year.

#### Bone complications

Bone complications were narrowly defined by the occurrence of osteonecrosis, pathological fractures, vertebral collapse, osteomyelitis, and/or bone crises/infarctions. A bone crisis was defined as an episode of severe pain localized in a bone (no joint), requiring opioids and/or hospitalization, and/or accompanied by signs of inflammation (e.g. fever), and/or imaging abnormalities (X-ray, magnetic resonance imaging). For most patients, bone complications had started long before their first presentation at the AMC. The exact date of onset of bone complications was recorded as precisely as possible. Again, if only the year in which a complication had occurred was recorded then the date was set for the first of January of that year.

#### Multiple complications

If a patient had experienced multiple bone complications separated by a symptom free interval, again according to the previously stated definitions, the date of their second bone complication was recorded. Parkinson disease and pulmonary hypertension were included as associated conditions in this phase.

#### Malignancy

Malignancies associated with Gaucher disease were defined as: multiple myeloma, AL amyloidosis, which is associated with monoclonal plasma cell proliferation or hepatocellular carcinoma. The starting date was chosen as the first occurrence of signs and/or symptoms associated with this condition.

#### Recovery

Date of resolution of signs and/or symptoms: unless explicitly stated otherwise, it was assumed that if signs and/or symptoms such as anemia had started prior to follow up at the AMC and were present at first presentation, they had been present in the meantime. Signs and/or symptoms were said to be resolved if:

1. Hb levels >13.5 g/dl (8.4 mmol/L) in men and >12 g/dl (7.5 mmol/L) in women (American Society of Hematology (ASH));

2. Platelet count was >100*10E9/L [31], for one year (if this was the case, then the date of the first measurement was recorded as the date of resolution, if follow-up ended before a year was reached then no date of resolution was recorded);

3. Liver volume was <1.25 multiples of normal (MN) and spleen volume was below 5 MN at two consecutive measurements [32] (no minimum for this interval was defined, in practice patients are evaluated 1–2 per year, the first measurement was recorded as the date of resolution);

4. A bone marrow fat fraction Ff as assessed by Dixon’s Quantitative Chemical Shift Imaging >23% at two consecutive measurements.

**Figure 1:**
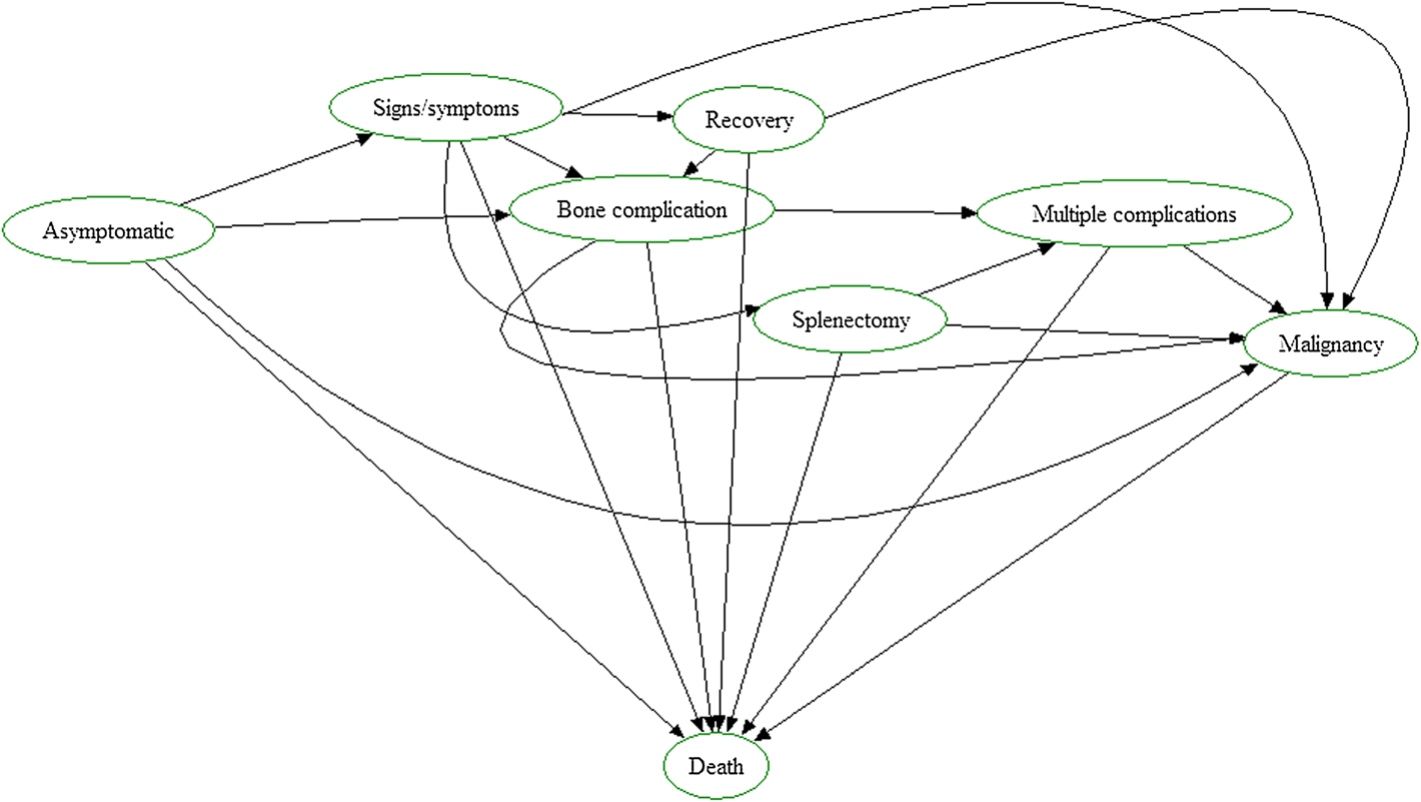
**Multi-state diagram of our proposed model.** Please note that this diagram represents both the NH chohort as well as the ERT cohort, with two exceptions: 1: recovery was not observed in the NH cohort. 2: the ERT cohort has no asymptomatic state as asymptomatic patients are not treated.

Figure 1 represents a multi-state diagram of our model. Please note that the corresponding model is depicted in Figure 1 of our manuscript on the cost-effectiveness of ERT for GD [30].

### Statistical analysis

Survival analysis was applied to study the time to progress to the next disease stage (e.g. from signs/symptoms to bone complications), while censoring patients at the end of their follow-up if they did not progress. It is generally assumed that censoring is non-informative. However, when patients may progress to one of multiple next disease stages (e.g. bone complications or splenectomy), then the patient may never progress to the disease stage of interest (bone complications), because of progressing to the other first (splenectomy), thus changing his probability of developing bone complications. If so, censoring for bone complications would be informative and entering the stage of splenectomy should be considered a competing risk event. The common Kaplan-Meier approach to survival analysis is not appropriate here [33]. Instead, a competing risk analysis was applied with data represented by a cumulative incidence curve for each next disease stage separately.

For consistency, data were also represented by cumulative incidence curves in absence of competing risks (including the 95% confidence intervals), except for an overall analysis of time till death in our cohort (irrespective of treatment status, treatment duration, and cause of death), when the usual Kaplan-Meier survival approach was applied.

A cumulative incidence curve was composed for the time to development of signs and/or symptoms in the NH cohort, with progression to bone complications as a competing risk.

Cumulative incidence survival curves were composed for both the NH and the ERT cohort for:

 time from signs and/or symptoms to a bone complication, with progression to splenectomy as competing risk

 time from signs and/or symptoms to (the need for) a splenectomy, with progression to a first bone complication as a competing risk

 time from a splenectomy to a bone complication (progression to the phase ‘multiple complications’)

 time from the first bone complication to progression to the phase ‘multiple complications’ (the occurrence of either a second bone complication or the need for a splenectomy), with progression to a malignancy as a competing risk

 time from signs and/or symptoms to recovery, with the progression to bone complications or a splenectomy as competing risks

**Figure 2:**
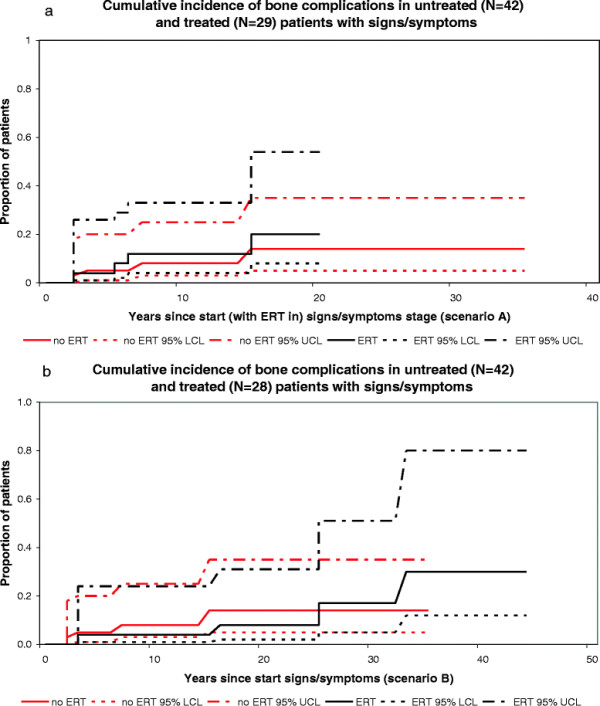
**Cumulative incidence of bone complications in untreated and treated patients with signs/symptoms. a**. scenario A, untreated N=42, treated N=29, **b**. scenario B, untreated (N = 42), treated (N = 28).

**Figure 3:**
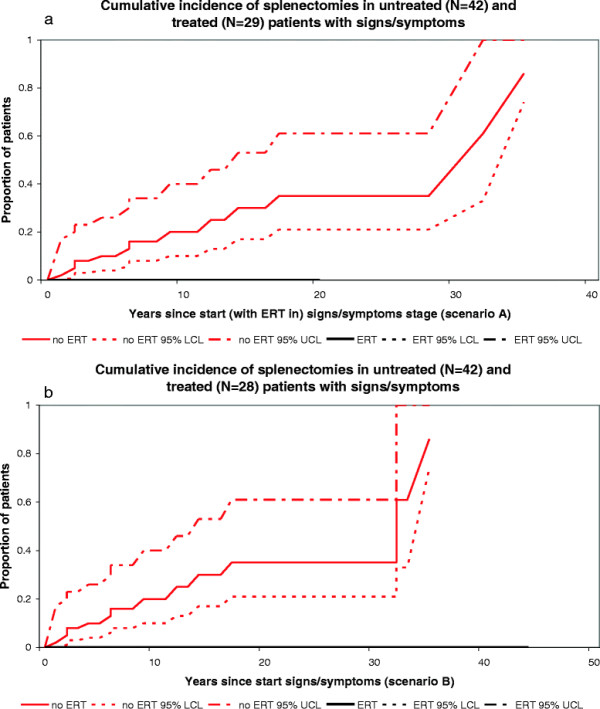
**Cumulative incidence of spenectomies in untreated and treated patients with signs/symptoms. a**. Scenario A, untreated N = 42, treated N = 29 **b**. Scenario B, untreated N = 42, treated N = 28.

**Figure 4:**
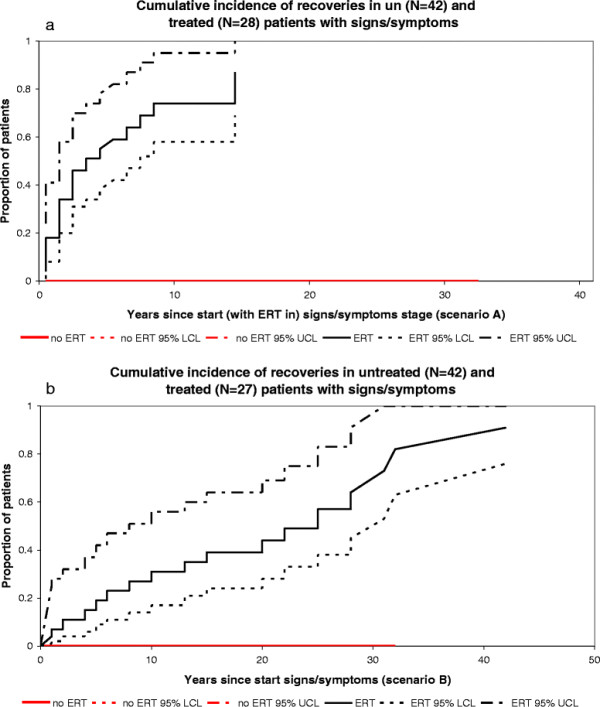
**Cumulative incidence of recoveries in untreated and treated patients with signs/symptoms. a**. Scenario A, untreated (N = 42), treated (N = 28). **b**. Scenario B, untreated N=42, treated N=27.

**Figure 5:**
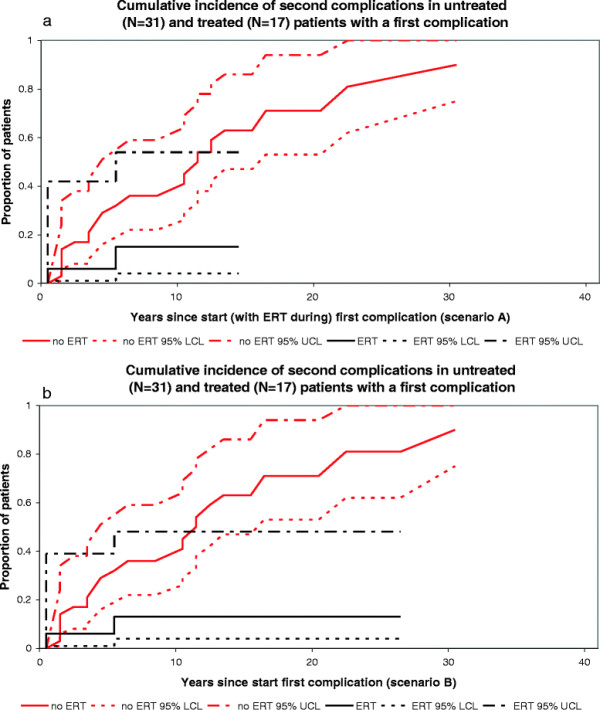
**Cumulative incidence of second complications in untreated and treated patients with a first complications. a**. Scenario A, untreated N=31, treated N=17 **b**. Scenario B, untreated N = 31, treated N = 17.

**Figure 6:**
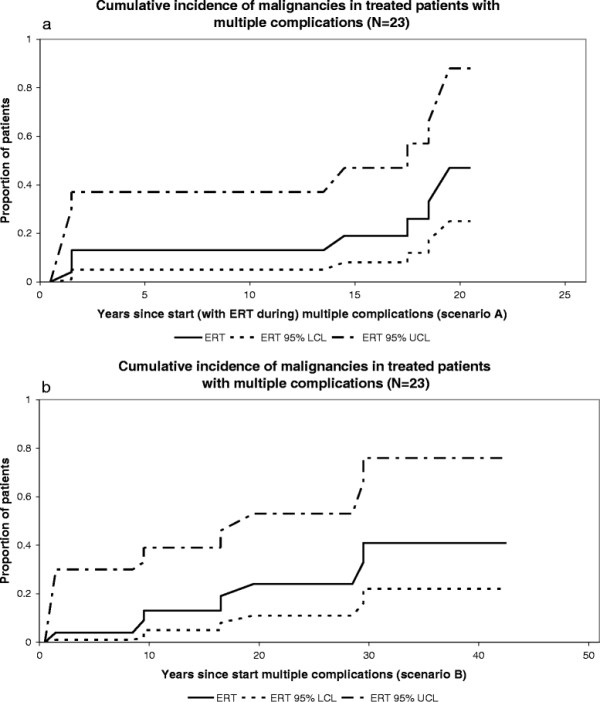
**Cumulative incidence of malignancies in treated patients with multiple complications. a**. Scenario A, N=23 **b**. Scenario B, N=23.

For the ERT cohort, cumulative incidence curves were drawn for 2 different scenarios. Since patients started treatment at different disease stages, the occurrence of a complication soon after start of treatment would not necessarily give a reliable estimate of the effect of therapy. To address this, the scenarios were as follows:

 In scenario A (depicted in Figures 2a, 3a, 4a, 5a and 6a), start of follow-up for each disease state was calculated from the date a patient started ERT.

 In scenario B (depicted in Figures 2b, 3b, 4b, 5b and 6b), the start of follow-up for each disease state was adjusted to the date a patient entered that stage (and not the date at which ERT was started), which means that some events took place at a later time point as compared to scenario A.

Patients in the signs/symptoms stage can recover as a result of ERT. For the analysis of progression from signs and/or symptoms to complications, the end of follow-up of these patients was adjusted to include the phase “recovery” and the stage that followed was adjusted to the stage that followed the phase “recovery”, i.e. if a patient recovered from signs and/or symptoms, but nonetheless developed a bone complication, the duration of follow-up in the signs/symptoms stage included both the signs/symptoms phase and the recovery phase and the patient was said to have experienced the event in the survival analysis for progression from signs and/or symptoms to bone complications.

Please note that this approach differs from the approach adopted in our accompanying paper on the cost-effectiveness analysis [30]. In this paper, “recovery” was treated as a separate stage in order to calculate the transition probabilities used in the Markov model. However, in the current paper all analyses are depicted as survival curves, which would lead to an overestimation of the chance of progression from “signs/symptoms” to “complications” as patients who recover are filtered out, leaving those patients who remain in the state “signs/symptoms” or those who experience a complication.

## Results

For ninety GD I patients clinical records or prospective data were available.

**Figure 7:**
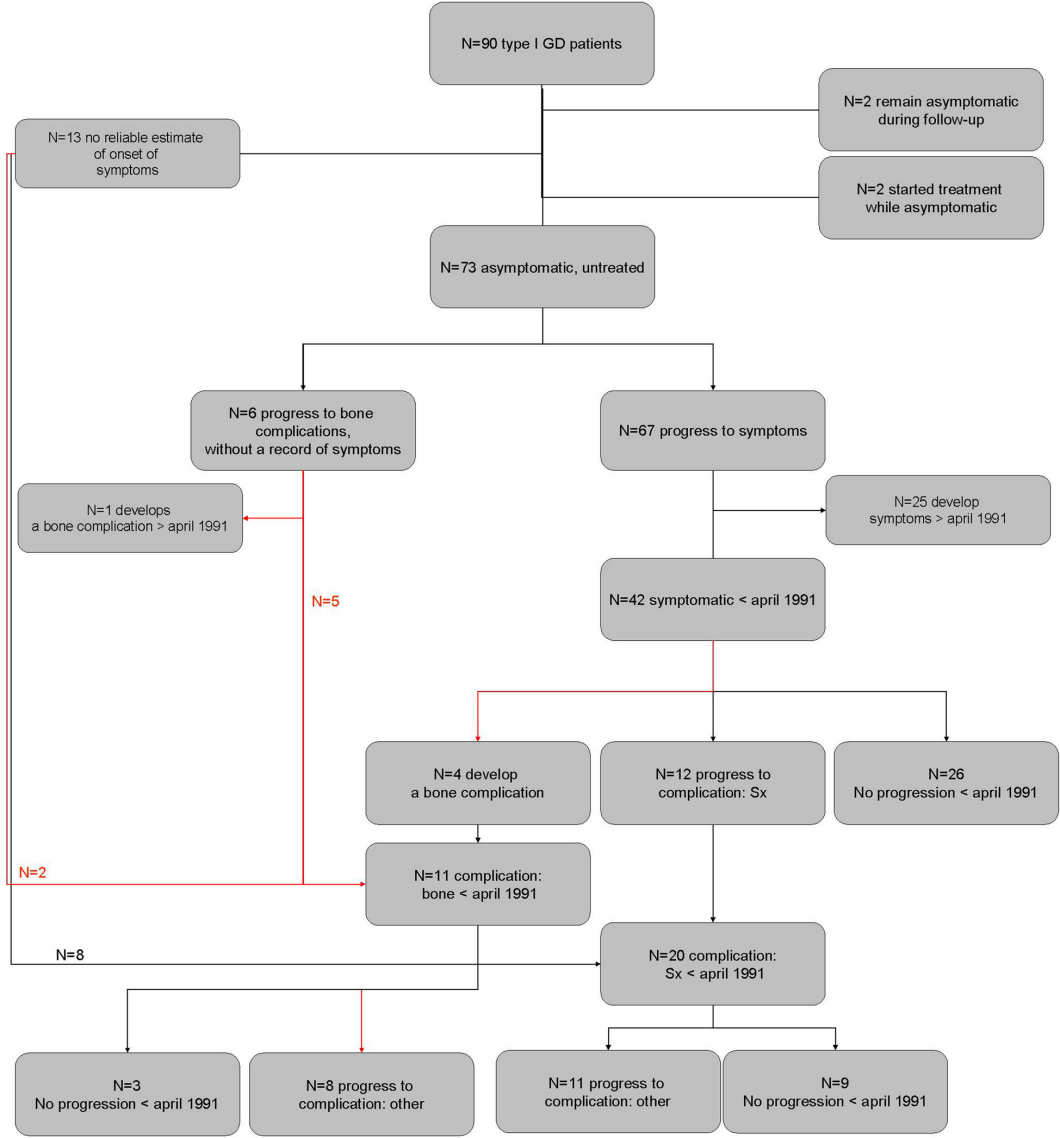
**Flow-chart of the NH cohort.** Please note that two patients who remained asymptomatic during follow-up were excluded from the analysis as both patient where diagnosed because of family studies. Two patients who started treatment while asymptomatic where excluded because the reasons/circumstances for starting treatment differed from other patients (e.g. patients’ wish).

**Figure 8:**
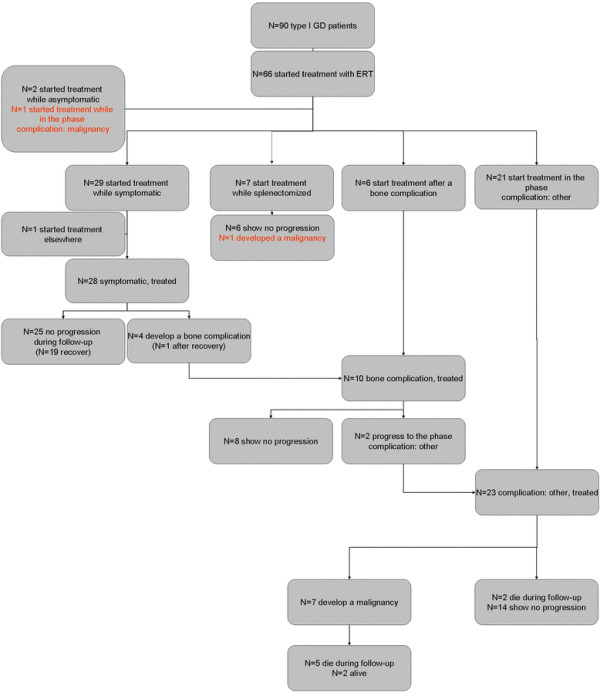
Flow-chart of the ERT cohort, start of follow-up = start ERT.

**Figure 9:**
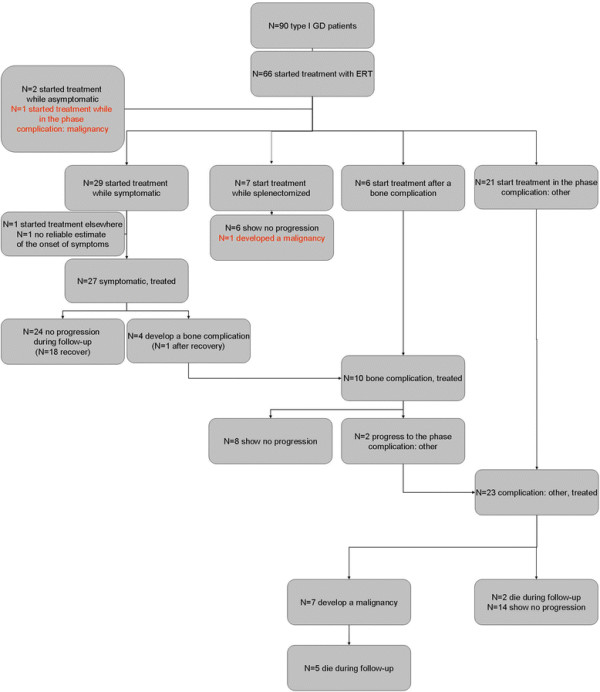
Flow-chart of the ERT cohort, start follow-up = start phase (in contrast to start ERT).

Figures 7, 8 and 9 represent the numbers of patients in each of the different disease stages for the NH and ERT cohort respectively. All percentages reported concern percentages derived from the survival analysis corrected for competing risks. All events that could be construed as competing risks for a specific analysis that occurred are mentioned below.

### Calculations of Progression or Recovery

#### Progression from asymptomatic to signs/symptoms

**Figure 10:**
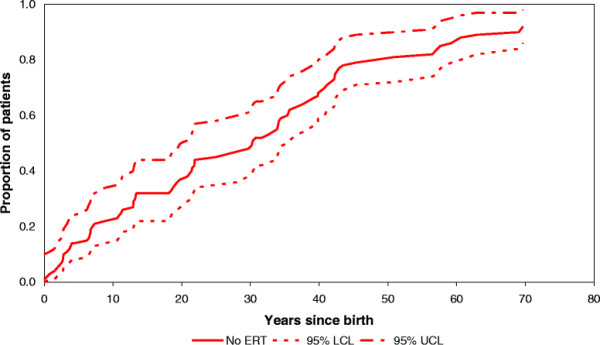
Cumulative incidence of signs/symptoms in asymptomatic patients (N = 73).

Of 90 patients, clinical records were available for 77 patients to determine the date of onset of signs and/or symptoms. Four patients did not qualify for the analysis of progression from asymptomatic to signs/symptoms: 2 patients who remained asymptomatic during follow-up and 2 patients who started treatment while they were asymptomatic. Of 73 asymptomatic patients, six developed a bone complication as the first disease manifestation and 67 developed cytopenia or organomegaly consistent with the predefined signs/symptoms stage. Median time to progression, corrected for bone complications as a competing risk was 30.1 years. Figure 10 depicts the cumulative incidence curve. No separate analysis for patients from the NH or ERT cohort is made, since asymptomatic patients are not eligible for treatment in the Netherlands.

#### Progression from signs/symptoms to complications, NH cohort

Of 67 patients, 42 had developed signs and/or symptoms prior to April 1991. Twenty-six patients showed no progression before April 1991, 4 developed a bone complication and 12 underwent a splenectomy. Nine percent had developed a bone complication after 10 years in the signs/symptoms phase, whereas 21 percent had undergone a splenectomy after 10 years in the signs/symptoms phase.

#### Progression from signs/symptoms to complications, ERT cohort

Twenty-nine patients started treatment in the signs/symptoms stage, i.e. before complications occurred.

Scenario A: of 29 patients, 25 showed no progression during follow-up. One patient died as a result of a renal cell carcinoma after recovery of signs/symptoms of Gaucher disease. Four patients with signs/symptoms developed a bone complication while treated. In one patient this bone complication occurred after recovery from signs/symptoms. Twelve percent had developed a bone complication after 10 years in the signs/symptoms phase. No patient in the ERT cohort underwent a splenectomy.

Scenario B: Of 28 out of the 29 above-mentioned patients a reliable record of onset of signs and/or symptoms was available. Twenty-four showed no progression during follow-up. One patient died as a result of a malignancy unrelated to Gaucher disease. Four patients with signs/symptoms developed a bone complication while treated. In one patient this bone complication occurred after she recovered from signs and/or symptoms. Four percent had developed a bone complication after 10 years in the signs/symptoms phase. No patient in the ERT cohort underwent a splenectomy while treated.

#### Progression from signs/symptoms to recovery, NH cohort

No patient in the NH cohort recovered.

#### Progression from signs/symptoms to recovery, ERT cohort

Twenty-nine patients started treatment in the signs/symptoms stage, i.e. before complications occurred. One patient had started treatment elsewhere and was transitioned to our center after he had recovered. The date of recovery could not be assessed in this patient.

Scenario A: of 28 patients, 19 recovered from signs and/or symptoms. Fifty percent had recovered after 3.6 years of treatment (median time to recovery).

Scenario B: of 27 out of the 28 above mentioned patients a reliable record of onset of signs and/or symptoms was available, 18 recovered from signs/symptoms. Fifty percent had recovered after 22.4 years.

Figures 2a-b and 3a-b represent the cumulative incidence curves for the NH and ERT cohort for progression from signs/symptoms to bone complications and splenectomy, respectively. Figure 4a-b show the cumulative incidence of recovery in the NH and ERT cohort.

#### Progression from a first complication to a second complication, NH cohort

Eleven patients had experienced a bone complication before April 1991. These include 4 patients who progressed from the signs/symptoms phase to bone complications and 5 patients who were diagnosed following a bone complication as the first manifestations of disease. In addition, 2 patients were diagnosed after a bone complication occurred and were found to display signs/symptoms at that time.

In eight patients a second complication occurred. Twenty patients were splenectomized before April 1991. In eleven patients a bone complication occurred before April 1991. Median time from the first to the second complication was 11 years.

#### Progression from a first complication to a second complication, ERT cohort

Thirteen patient started treatment after a first complication had occurred (N = 7 after a splenectomy, N = 6 after a bone complication). In addition four patients had developed a bone complication while treated.

Of 10 patients who had experienced a single bone complication and received treatment, 8 showed no progression and 2 developed a second complication.

Of seven patients who started treatment after having been splenectomized, 1 patient developed a malignancy while treated (a hepatocellular carcinoma), 6 patients showed no progression.

One patient developed pulmonary hypertension, while already in the phase multiple complications. Signs of pulmonary hypertension had been present before the start of ERT. Another patient was diagnosed with Parkinson disease while already in the phase multiple complications. This patient had been receiving ERT for more than 13 years at the time of the diagnosis Parkinson disease.

Figures 5a-6 depict the cumulative incidence curves from a first complication to the phase ‘multiple complications’.

In scenario A sixteen percent had experienced a second complication, 10 years after a first complication.

In scenario B fourteen percent had experienced a second complication 10 years after a first complication had occurred.

#### Malignancy

Nine patients in the entire Dutch cohort had developed a Gaucher associated malignancy. Characteristics of these patients are included in Table 1. One patient was treatment naive when a diagnosis of multiple myeloma was made, 3.1 years after a splenectomy had been performed. Since this diagnosis was made after April 1991 this case was excluded from the NH cohort. As previously described, one patient developed a hepatocellular carcinoma 22.4 years after a splenectomy (6.1 years after start of ERT).

**Table 1:** Characteristics of 9 GD patients who developed a GD associated malignancy

**Complication**	**Treatment status at diagnosis**	**Disease phase at diagnosis**	**Genotype**
Amyloidosis/MM	ERT <2 years	Multiple complications	N370S/L444P
HCC	ERT <2 years	Multiple complications	N370S/I260T
Amyloidosis/MM	untreated	Complication: Sx	N370S/L444P
HCC	ERT 6 years	Complication: Sx	R463C/?
Amyloidosis/MM	ERT 2 years	Multiple complications	N370S/R257Q
Amyloidosis/MM	ERT 15 years	Multiple complications	N370S/L444P
HCC	ERT >17 years	Multiple complications	N370S/L324P
Amyloidosis/MM	ERT 19 years	Multiple complications	N370S/G84GG
HCC	ERT 18 years	Multiple complications	N370S/N370S

Seven patients developed an associated malignancy after having experienced multiple Gaucher related complications; 3 patients developed a hepatocellular carcinoma (all were splenectomized) and four patients developed a multiple myeloma (of whom 2 were splenectomized). Figure 6a-b depict the cumulative incidence curves for progression from multiple complications to malignancy.

In scenario A fourteen percent had developed a malignancy 10 years after having started treatment in the phase multiple complications.

In scenario B fourteen percent had developed a malignancy 10 years after their second complication.

**Figure 11:**
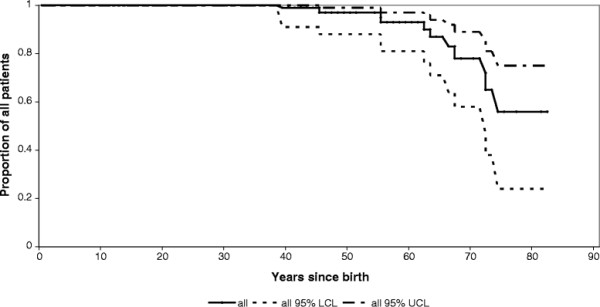
**Cumulative survival in the entire study cohort, irrespective of treatment status, treatment duration, and cause of death (N = 90).** Red lines represent the NH cohort, black lines represent the ERT cohort. LCL: lower confidence limit. UCL: upper confidence limit.

A survival curve of the entire study cohort, irrespective of treatment status is presented in Figure 11.

## Discussion and conclusion

Numerous studies have shown that enzyme replacement therapy for Gaucher disease is effective in reversing signs/symptoms such as cytopenia and organomegaly (see for example [15]-[21]). Studies have also shown that the effects on signs/symptoms are accompanied by improvements in quality of life (QoL) [23]-[27]. The study by Damiano et al. shows that a history of splenectomy and joint replacement was associated with a decrease in QoL. The study by Giraldo et al. supports this, but did not find an association between degree of cytopenia and QoL. Given this high impact of long-term complications and associated conditions of Gaucher disease on QoL, it is important to investigate the effect of ERT on the incidence of these events. In addition, from a cost-effectiveness perspective, the question whether ERT can prevent morbidity is valid as well, since treatment is very expensive.

As expected, a large proportion of ERT treated patients recovered from a signs/symptoms state to an asymptomatic state. None of the patients in our historic NH cohort recovered spontaneously. We have reported earlier that mildly affected patients may show some degree of spontaneous improvement [34], but for the more severely affected NH cohort patients in the current study, an improvement that would reclassify them to the asymptomatic stage was not observed.

Progression from the signs/symptoms stage to the development of complications was positively influenced by ERT. First, we studied the effect of enzyme replacement therapy on the need for a splenectomy. Severe cytopenia due to functional hypersplenism and mechanical pressure causing abdominal complaints may both necessitate a splenectomy. Since ERT has been shown to effectively reduce splenomegaly and cytopenia one might assume that splenectomies in the ERT era are rarely indicated. Indeed, our study shows significant differences in the incidence of this complication in the NH cohort when compared to the ERT cohort. In fact, none of the treated patients underwent a splenectomy. However, there are anecdotal reports to suggest that ERT might not be able to prevent the need for a splenectomy in rare circumstances such as the development of neutralizing antibodies to ERT (Ponce et al. 1997) or extensive fibrosis (Krasnewich et al. 1998) rendering a patient irresponsive to treatment [35],[36].

Bone complications are another important group of (long-term) complications in GD I that have an important impact on the quality of life of patients [23],[24],[27]. The effect of ERT on bone complications is slightly more controversial. Several studies show that ERT improves imaging parameters thought to reflect a patient’s risk of developing bone complications. These include the Rosenthal staging system [37], Quantitative Chemical Shift Imaging (QCSI) measurements [37]-[39], the Düsseldorf marrow disease score [40], and Bone Marrow Burden (BMB) scores [41]. The effect of ERT on bone mineral density measurements is more controversial [42]-[45]. While an effect of ERT on these markers is important to note, clearance of Gaucher cells from the bone marrow does not necessarily mean bone metabolism is restored [46], although it is likely that in previously asymptomatic patients, clearance of Gaucher cells from the bone marrow poses less risk to patients for development of bone complications [47]. Other studies have attempted to quantify the effects of ERT on clinical bone disease describing positive effects on bone pain and/or a reduction in the frequency of bone events in ERT treated patients [48]-[52]. These studies are supported by data from the Gaucher Registry [53],[54] including a recent study by Mistry et al., which shows that patients who initiated ERT within 2 years of diagnosis had a significantly reduced risk of developing osteonecrosis compared to those who started more than 2 years after their diagnosis [55]. This may not seem surprising as the latter group consisted of patients with more pretreatment bone disease, developed before ERT was available. The data presented here are derived from the entire Dutch cohort and the natural history data were carefully selected to avoid limitations such as bias by indication. It offers further evidence that ERT can significantly reduce the frequency of bone complications, but cannot fully prevent these. The two scenarios that were employed show the largest difference for the occurrence of bone complications: 12% or 4% within 10 years of ERT for scenario A and B respectively. Within scenario A, patients could develop a bone complication quite quickly after start of ERT, as their time to complication was calculated from start of ERT. It is questionable whether this gives a realistic estimate of the effect of ERT. To overcome this, we calculated the time between the occurrence of a complication and the start of the previous disease state. This may give a more reliable estimate of the time to develop a complication, although the most optimal effect of ERT to reduce the occurrence of bone complications can only be assessed in a situation where only early symptomatic patients, without a history of bone complications, enter the model.

Finally, we studied the incidence of Gaucher associated malignancies. These malignancies occurred almost exclusively in the ERT treated patients. However, associated malignancies are certainly not a consequence of treatment as evidenced by numerous studies and case reports describing the occurrence of these malignancies in untreated patients (see for example Lee et al. [56]). The reason for the absence of malignancies in the natural history cohort is obviously caused by the fact that clinical records were only available from patients who had visited our center at least once after April 1991, and those with associated malignancies had probably died earlier. Our data illustrate that malignancies occur mostly in patients with advanced Gaucher disease, which would have provided patients with a clear indication to start treatment. Our study is thus subject to a certain selection bias, as patients with associated malignancies are underrepresented in our NH cohort, which might underestimate the effect of ERT. Furthermore, age is an important determinant of cancer risk. Our treated cohort consists largely of patients from the NH cohort that have aged and this will have influenced cancer incidence. By showing that ERT effectively prevents/reduces progression to more advanced stages of disease, it is suggested that ERT is able to reduce the frequency of Gaucher associated malignancies. This is supported by the observations that: 1) hepatocellular carcinoma in GD I patients is exclusively seen in splenectomized patients and splenectomies were only performed in untreated patients, and 2) only one patient developed an MGUS, while treated. MGUS in itself is not a malignancy, but in the general population the yearly risk of transition from an MGUS to a multiple myeloma or other lymphatic malignancy is 1% [57].

However, some caution with regard to the effect of ERT on malignancies is warranted as they represent a heterogeneous group. While the occurrence of HCC has thus far only been reported in patients with advanced GD [9], multiple myeloma has been reported in patients displaying mild disease (i.e. [58]) and its association with disease severity or extent of residual disease may be less straightforward.

Furthermore, with regard to the hepatocellular carcinoma, while recent evidence suggests an increased risk in GD patients [9],[59], literature on its pathophysiology is limited and this aspect deserves further study [10].

The retrospective design of this study is an important limitation. Data on signs/symptoms and complications and associated conditions may not have been recorded as rigorously as would have been the case in a prospectively designed study. Consequently, the rate of disease progression in untreated patients has possibly been underestimated in the present analysis. At the same time, disease progression among patients receiving ERT has probably been overestimated, because many patients already had spent some time in the disease stage before ERT became available on the market in 1991; this delayed start with ERT may have resulted in suboptimal treatment. This is a particular limitation for scenario A, in which previous time spent in the disease state is ignored. Therefore scenario B was developed in which this time was added to the time on treatment in the specific disease stage. However, this scenario may still lead to an overestimation of the rate of disease progression under ERT since ERT has in fact not been given during the entire disease stage. On the other hand, both scenarios are subject to “immortal time bias”: only patients who were alive and in the disease state under study at or after the introduction of ERT are included, which may result in an underestimation of disease progression. While both scenario’s are artificial, these effects may balance each other to some extent. Statistical approaches with ERT use as a time-varying covariate (e.g. Cox regression) would also be worth studying in order to assess the impact of potential misclassification and selection biases.

Given the variations in dosing regimens worldwide, it is of interest to discuss the effect of ERT dose on disease progression and incidence of long-term complications and associated conditions. A previous study showed that high dose ERT results in faster and more pronounced responses in plasma chitotriosidase activity and bone marrow involvement [60]. A subsequent study from the Gaucher Registry confirmed a dose effect on clinical parameters [61]. One might hypothesise that high dose ERT results in a more effective prevention of long-term complications and associated conditions. However, the relationship between the extent of residual disease and the risk for long-term complications and associated conditions remains unclarified.

In conclusion, long-term enzyme replacement therapy for Gaucher disease can effectively reduce the incidence of splenectomy and bone complications, and will most likely result in a reduction in the risk of developing malignancies

## Competing interests

The submitted work was supported only by Top Institute Pharma; CH has received reimbursement of expenses and honoraria for lectures on the management of from Genzyme, Actelion, Protalix and Shire HGT. MB has received reimbursement of expenses and honoraria for lectures on the management of from Genzyme, Actelion and Shire HGT. MB and CH donated the honoraria to the Gaucher Stichting, a foundation that supports research in the field of lysosomal storage disorders. LvD has received travel reimbursements at two occasions (Genzyme, Protalix).

MD has no relationships with companies that might have an interest in the submitted work in the previous three years; none of the authors has non-financial interests that may be relevant to the submitted work.

## Authors’ contributions

LD analysed the data and wrote the manuscript. MD advised and supervised the statistical methods adopted. MB, MD and CH critically revised the manuscript. All authors read and approved the final manuscript.
